# Typhus Fever in New-York

**Published:** 1847-06

**Authors:** 


*Typhus Fever in New-York.*—We learn that Typhus Fever prevails extensively among the newly arrived Emigrants from Ireland. It is said to present the distinguishing traits of true Typhus. Several hundred cases are reported to have been received at the Bellevue Hospital. The same disease is now prevailing extensively in Dublin, and throughout Ireland.

